# Supplementary material for: Multi‐dataset identification of innovative feature genes and molecular mechanisms in keratoconus
Source: J Cell Mol Med. 2024 Sep 19;28(18):e70079. doi: 10.1111/jcmm.70079 (PMC11412914; doi:10.1111/jcmm.70079)
Supplement: Supplementary file 2 — Material S2. [file JCMM-28-e70079-s002.zip › 7.GSEA analysis/3.DBNDD1/KEGG_SPHINGOLIPID_METABOLISM.html]

Details for gene set KEGG\_SPHINGOLIPID\_METABOLISM[GSEA]

|  || Dataset | DBNDD1.group.cls#h\_versus\_l |
| Phenotype | group.cls#h\_versus\_l |
| Upregulated in class | l |
| GeneSet | KEGG\_SPHINGOLIPID\_METABOLISM |
| Enrichment Score (ES) | -0.33026358 |
| Normalized Enrichment Score (NES) | -1.1504499 |
| Nominal p-value | 0.27128714 |
| FDR q-value | 0.6991734 |
| FWER p-Value | 0.995 |
Table: GSEA Results Summary

  

Fig 1: Enrichment plot: KEGG\_SPHINGOLIPID\_METABOLISM      
 Profile of the Running ES Score & Positions of GeneSet Members on the Rank Ordered List

  

| SYMBOL | TITLE | RANK IN GENE LIST | RANK METRIC SCORE | RUNNING ES | CORE ENRICHMENT || 1 | UGT8 | na | 736 | 0.410 | 0.1040 | No |
| 2 | ACER1 | na | 3591 | 0.228 | 0.0913 | No |
| 3 | ACER2 | na | 6407 | 0.139 | 0.0527 | No |
| 4 | NEU3 | na | 10403 | 0.066 | -0.0423 | No |
| 5 | SGPL1 | na | 10503 | 0.064 | -0.0255 | No |
| 6 | ENPP7 | na | 11076 | 0.057 | -0.0245 | No |
| 7 | NEU4 | na | 11727 | 0.050 | -0.0280 | No |
| 8 | B4GALT6 | na | 12863 | 0.039 | -0.0487 | No |
| 9 | SPHK1 | na | 13199 | 0.037 | -0.0472 | No |
| 10 | KDSR | na | 13268 | 0.036 | -0.0382 | No |
| 11 | DEGS2 | na | 13783 | 0.032 | -0.0433 | No |
| 12 | SGPP1 | na | 15556 | 0.019 | -0.0886 | No |
| 13 | ASAH2 | na | 15878 | 0.017 | -0.0928 | No |
| 14 | ARSA | na | 16465 | 0.013 | -0.1057 | No |
| 15 | SPTLC2 | na | 16973 | 0.010 | -0.1174 | No |
| 16 | SGMS1 | na | 18281 | 0.001 | -0.1549 | No |
| 17 | GALC | na | 18285 | 0.001 | -0.1547 | No |
| 18 | GLB1 | na | 18534 | -0.001 | -0.1615 | No |
| 19 | ACER3 | na | 18589 | -0.001 | -0.1626 | No |
| 20 | SPTLC1 | na | 19710 | -0.010 | -0.1917 | No |
| 21 | ASAH1 | na | 20310 | -0.016 | -0.2042 | No |
| 22 | SMPD2 | na | 21053 | -0.023 | -0.2187 | No |
| 23 | NEU1 | na | 21377 | -0.026 | -0.2202 | No |
| 24 | SMPD4 | na | 22526 | -0.037 | -0.2418 | No |
| 25 | CERK | na | 25596 | -0.080 | -0.3058 | Yes |
| 26 | SMPD1 | na | 26047 | -0.088 | -0.2917 | Yes |
| 27 | GLA | na | 26169 | -0.091 | -0.2674 | Yes |
| 28 | DEGS1 | na | 28029 | -0.132 | -0.2807 | Yes |
| 29 | SPHK2 | na | 28315 | -0.138 | -0.2466 | Yes |
| 30 | GAL3ST1 | na | 28773 | -0.151 | -0.2137 | Yes |
| 31 | SGMS2 | na | 29604 | -0.176 | -0.1839 | Yes |
| 32 | UGCG | na | 29898 | -0.185 | -0.1356 | Yes |
| 33 | SMPD3 | na | 30792 | -0.214 | -0.0960 | Yes |
| 34 | SGPP2 | na | 31596 | -0.245 | -0.0444 | Yes |
| 35 | NEU2 | na | 34086 | -0.441 | 0.0185 | Yes |
Table: GSEA details [plain text format]

  

Fig 2: KEGG\_SPHINGOLIPID\_METABOLISM      
 Blue-Pink O' Gram in the Space of the Analyzed GeneSet

  

Fig 3: KEGG\_SPHINGOLIPID\_METABOLISM: Random ES distribution      
 Gene set null distribution of ES for **KEGG\_SPHINGOLIPID\_METABOLISM**
